# Remarks on Malignant Fevers; and Their Cure by Cold Water and Fresh Air

**Published:** 1786

**Authors:** William Wright

**Affiliations:** Fellow of the Royal College of Physicians at Edinburgh, and one of the Physicians General in Jamaica


### LONDON MEDICAL JOURNAL,

FOR THE YEAR 1786,

### PART THE SECOND.

I. *Remarks on Malignant Fevers; and their Cure by cold Water and fresh Air. Communicated in a Letter to* Samuel Foart Simmons, *M. D. F. R. S. by* William Wright, *M. D F. R. S. Fellow of the Royal College of Physicians at Edinburgh, and one of the Physicians General in Jamaica.*

FROM the time that physicians have found fresh air and cold watery drinks so beneficial in the small-pox and malignant fevers, these diseases have been less fatal within the tropics than formerly.

Having often observed how greatly people, labouring under malignant fevers, were refreshed by washing the hands and face in cold water, I was led to think that the cold bath would answer many good purposes in obstinate, malignant, and putrid fevers; but a practice so new in Jamaica, and so different from the common methods, could not well be propofed ; and if it had, would probably not have been fubmitted to : on which account, I kept my opinion to myfelf till fome favourable opportunity ; which did not happen till I was on my paffage from Jamaica to England.

On the 1ft of Auguft, 1777, I embarked in a fhip bound to Liverpool, and failed the fame evening from Montego Bay. The mafter told me he had hired feveral failors on the fame day we took our departure ; one of whom had been long at fick quarters on fhore, and was now but in a convalefcent ftate.

Auguft 23d, we were in the latitude of Bermudas, and had had a heavy gale of wind for three days, when the above-mentioned man relapfed, and had a fever, with fymptoms of the greateft malignity. I attended this perfon often, but could not prevail with him to be removed from a dark and confined fituation to a more airy and convenient part of the fhip ; and as he refufed medicines, and even food, he died on the eighth day of his illnefs.

By my attention to the fick man, I caught the contagion, and began to be indifpofed on the 5th of September, and the following is a narrative of my own cafe, extracted from notes daily daily marked down: — I had been many years in Jamaica, but, except being ſomewhat relaxed by the climate and fatigue of buſineſs, I ailed nothing when I embarked. This circumſtance, however, might, perhaps, diſpoſe me more readily to receive the infection.

September 5th, 6th, 7th, ſmall rigors now and then — a preternatural heat in the ſkin — a dull pain in the forehead — the pulſe ſmall and quick — a loſs of appetite, but no ſickneſs at ſtomach — the tongue white and ſlimy — little or no thirſt — the belly regular — the urine pale and rather ſcanty — in the night reſtleſs, with ſtartings and delirium.

September 8th, every ſymptom aggravated, with pains in the loins and lower limbs, and ſtiffneſs in the thighs and hams.

I took a gentle vomit on the ſecond day of this illneſs, and next morning a decoction of tamarinds; at bed-time, an opiate, joined with antimonial wine, but this did not procure ſleep, or open the pores of the ſkin. No inflammatory ſymptoms being preſent, a drachm of Peruvian bark was taken every hour for ſix hours ſucceſſively, and now and then a glaſs of port wine, but with no apparent benefit. When upon deck, my pains were greatly mitigated, and the colder the air the better. This circumſtance, and the failure of every means I had tried, encouraged me to put in practice on myſelf what I had often wiſhed to try on others in fevers ſimilar to my own.

September 9th, having given the neceſſary directions, about three o'clock in the afternoon I ſtripped off all my cloaths, and threw a ſea cloak looſely about me till I got upon deck, when the cloak alſo was laid aſide: three buckets full of cold ſalt water were then thrown at once on me; the ſhock was great, but I felt immediate relief. The head-ach and other pains inſtantly abated, and a fine glow and diaphoreſis ſucceeded. Towards evening, however, the febrile ſymptoms threatened a return, and I had recourſe again to the ſame method, as before, with the ſame good effect. I now took food with an appetite, and, for the firſt time, had a ſound night's reſt.

September 10th, no fever, but a little uneaſineſs in the hams and thighs—uſed the cold bath twice.

September 11th, every ſymptom vaniſhed, but, to prevent a relapſe, I uſed the cold bath twice.

Mr.

Mr. Thomas Kirk, a young gentleman, paſſenger in the ſame ſhip, fell ſick of a fever on the 9th of Auguſt. His ſymptoms were nearly ſimilar to mine, and, having taken ſome medicines without experiencing any relief, he was deſirous of trying the cold bath, which, with my approbation, he did on the 11th and 12th of September, and, by this method, was happily reſtored to health. He lives at this time near Liverpool.

There are a number of teſtimonies, both ancient and modern, of the cure of putrid and malignant fevers by adminiſtering cold water, in large quantities, for common drink, and applying cold water externally to the ſurface of the body.

The Greek phyſicians extinguiſhed the intenſe heat of ardent fevers, at their height, by making their patients drink large quantities of cold water, and ſometimes plunging them into a cold bath. A copious and critical ſweat was always expected to follow this practice.

Dr. Cyrillus *, a learned and ingenious phyſician and profeſſor at Naples, has favoured us with a circumſtantial account of the good effects

* Phil. Tranſ. Vol. XXXVI. No. 410.

of of cold water given internally in malignant fevers at Naples, and obferves, that, in obftinate cafes, powdered fnow was laid on the breafts of the fick. The fuccefs of this mode was fuch, that this practice was univerfally adopted there, and ftill continues to the prefent time.

Dr. J. G. de Hahn has given* us the hiftory of a putrid epidemic, which prevailed at Breflaw in 1737, and in which every method of cure was ineffectual, till cold water was applied with fponges to the whole furface of the body. Among other proofs of the efficacy of this mode of treatment, the author mentions its fuccefsful ufe in his own cafe.

Sir John Chardin, when at Gambroon in 1673, was feized with a malignant, burning fever, attended with delirium and many other bad fymptoms; and of which, after having had many medicines prefcribed without the defired effect, he was fpeedily cured by the cold bath.

" This uncommon and furprifing practice, " (fays Dr. Glafs †) fo fuccefsfully employed

* *Vide* Acta Phyfico-Medica Acad. Nat. Cur. Vol. X. p. 3. 4to. Norimbergæ, 1754.

† See Dr. Glafs's firft letter to Dr. Baker, page 37. 8vo. London, 1767.

" in

"in curing a burning fever, accompanied
"with weaknefs, faintnefs, and proftration of
"ftrength, without any apparent caufe, when
"duly confidered, points out a more fuccefsful
"method of treating our putrid, malignant
"fevers than that which is at prefent moft
"commonly ufed."

In the Weft Indies fevers are lefs contagious than in this country, becaufe the fame caufes of contagion do, in general, not exift there; the fick being placed in airy and well-ventilated chambers: but in jails, in crowded hofpitals and fhips, fevers, in the Weft Indies, are as infectious as in Europe; of which I have feen many examples within thefe laft four years. The cure, in thofe cafes, was effected by a removal of the fick to better air — by cleanlinefs in apparel and bed-cloaths — frequent bathing in the fea for a fhort time — cold water alone for drink, or acidulated with elixir of vitriol — a moderate ufe of wine — and the bark.

*London,*
January 2, 1786.

